# Association Between *ERCC1* rs3212986 and *ERCC2/XPD* rs1799793 and OS in Patients With Advanced Esophageal Cancer

**DOI:** 10.3389/fonc.2019.00085

**Published:** 2019-02-21

**Authors:** Elisa Boldrin, Sandro Malacrida, Enrica Rumiato, Giorgio Battaglia, Alberto Ruol, Alberto Amadori, Daniela Saggioro

**Affiliations:** ^1^Immunology and Molecular Oncology, Veneto Institute of Oncology IOV-IRCCS, Padova, Italy; ^2^Eurac Research, Institute of Mountain Emergency Medicine, Bolzano, Italy; ^3^Endoscopy Unit, Veneto Institute of Oncology IOV-IRCCS, Padova, Italy; ^4^Department of Surgical Sciences, Oncology and Gastroenterology, University of Padova, Padova, Italy

**Keywords:** esophageal cancer, overall survival, germline variants, *ERCC2/XPD* rs1799793, *XPD* Asp312Asn, *ERCC1* rs3212986, *ERCC1* C8092A

## Abstract

Esophageal cancer (EC) is a very aggressive tumor, and no reliable prognostic markers exist especially for resectable advanced neoplasia. The principal aim of this study was to investigate the association of germline polymorphisms in nucleotide excision repair (NER) pathway genes with the overall survival (OS) of patients with advanced EC. As a second aim, we also studied the association of NER gene variants with response to cisplatin-based chemotherapy. Among the EC patients referred to our Institution between 2004 and 2012, we selected a cohort of 180 patients diagnosed with a clinical tumor stage ranging from IIB and IVA. Patients were genotyped for four NER variants, two in the *ERCC1* (rs11615 and rs3212986) and two in the *ERCC2/XPD* (rs1799793 and rs13181) genes. Kaplan–Meier analyses and Cox proportional hazards model were used to evaluate the associations of the selected variants with OS; association with response to neoadjuvant therapy was investigated using logistic regression. Results showed that the *ERCC1* rs3212986 and the *ERCC2/XPD* rs1799793 were significantly associated with shorter OS. On the contrary, response association analysis displayed that, while rs11615 and rs3212986 in *ERCC1* were associated with response, both *ERCC2/XPD* variants were not. By creating survival prediction models, we showed that the rs3212986 and the rs1799793 have a better predictability of the tumor stage alone. Furthermore, they were able to improve the power of the clinical model (AUC = 0.660 vs. AUC = 0.548, *p* = 0.004). In conclusion, our results indicate that the *ERCC1* rs3212986 and the *ERCC2/XPD* rs1799793 could be used as surrogate markers for a better stratification of EC patients with advanced resectable tumor.

## Introduction

Esophageal cancer (EC) is a highly lethal malignancy, usually diagnosed at an advanced stage (1). Surgery is the standard of care for potentially resectable neoplasia, and very often is preceded by a cisplatin-based neoadjuvant chemotherapy. The two predominant histologic subtypes are adenocarcinoma (EADC) and squamous cell carcinoma (ESCC). EADC is thought to arise from an acquired precursor condition known as Barrett's esophagus caused by chronic gastro-esophageal reflux (2, 3). Other EADC risk factors, also shared by ESCC, include tobacco and alcohol consumption, habits that lead to a chronic inflammation status (4).

Epidemiological studies have shown that chronic inflammation predisposes to different types of cancer and, for this reason, inflammation has been proposed as the seventh hallmark of cancer (5). One of the mechanism involved in cancer-related inflammation is the induction of genetic instability resulting in random DNA alterations (5, 6). DNA repair genes are crucial for the maintenance of the integrity of DNA damaged by both endogenous and exogenous hazardous agents. Thus, DNA repair genes and their constitutive variants have been indicated as a possible cause of the inter-individual variability to chemotherapy and patient outcome or as a factor that could modify the risk of tumor development (7–14).

Among the different DNA repair mechanisms, nucleotide excision repair (NER) is one of the most relevant pathway involved in the repair of DNA damaged by tobacco, radiation, free radicals, and chemotherapeutic agents (15). Excision repair cross-complementing 1 (ERCC1) and excision repair cross-complementing 2/xeroderma pigmentosum group D (ERCC2/XPD) are considered two key rate-limiting enzymes in the multistep NER process, because of their pivotal role in the recognition and removal of damaged nucleotides. Both *ERCC1* and *ERCC2/XPD* have single nucleotide polymorphisms (SNPs) that modulate their DNA repair capability (16–19). So far, numerous studies have investigated the potential predictive/prognostic power of constitutive genetic variants of these repair genes in a wide range of neoplasia, including EC, with contradictory results (20–24).

The first aim of this study was to define a possible association between variants in *ERCC1* (rs11615 and rs3212986), and *ERCC2/XPD* (rs13181 and rs1799793) genes and OS of patients with advanced resectable EC; as a second aim, we also evaluated the association of the same genetic variants with response to cisplatin-based neoadjuvant chemotherapy. To restrain possible bias, among all the EC patients referred to our Institute between 2004 and 2012, we selected a cohort of patients, whose OS was not significantly affected by their clinico-pathological characteristics. The rs11615 (Asn118Asn) and rs3212986 (C8092A) in *ERCC1*, and the rs13181 (Lys751Gln) and rs1799793 (Asp312Asn) in *ERCC2/XPD* were selected based on their putative association with altered DNA repair capability (16–19), and their high frequency in the Caucasian population (minor allele frequency ranging from 28 to 42%).

## Materials and Methods

### Study Endpoints and Patient Selection

**Figure 1:**
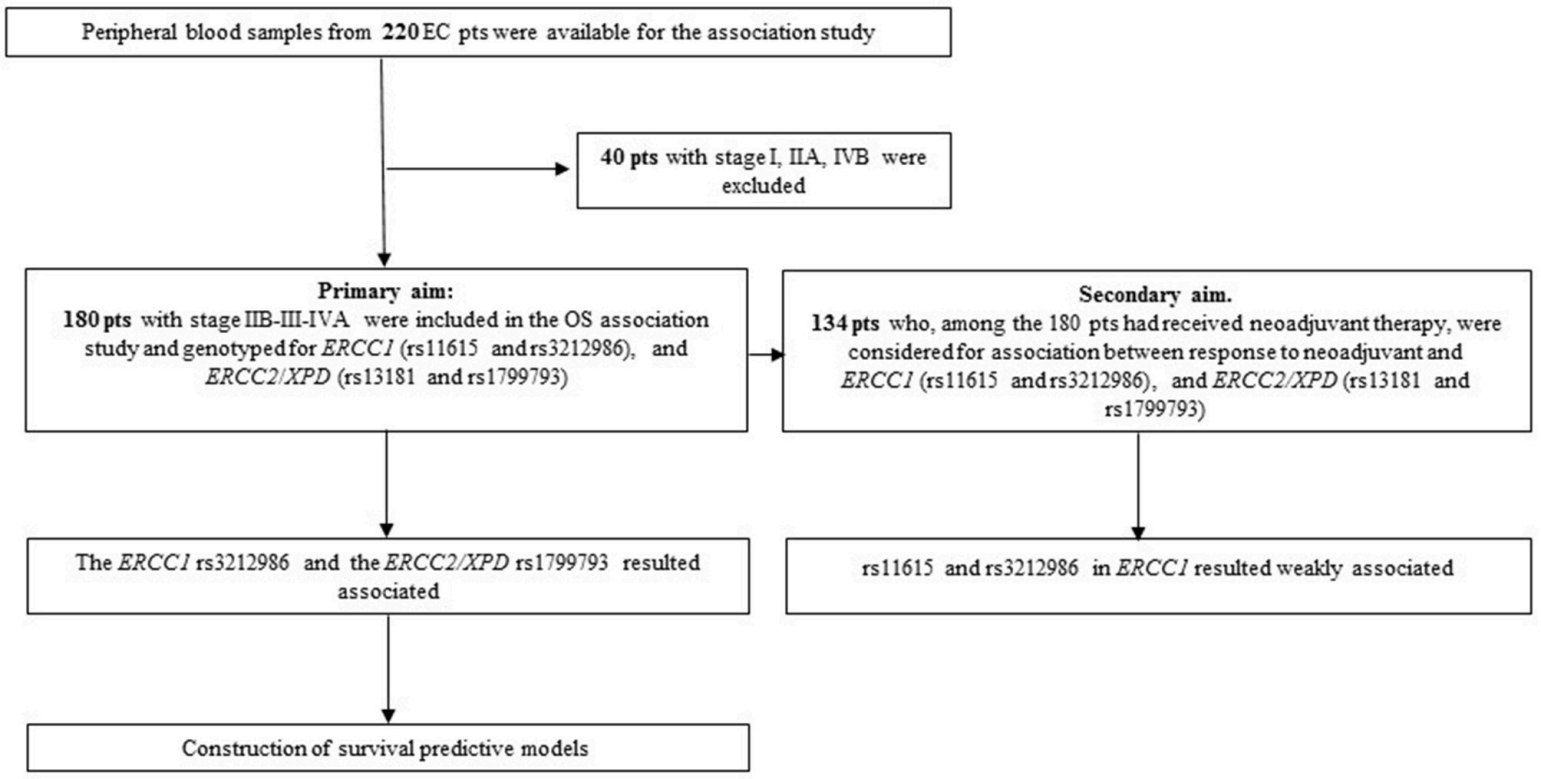
Flowchart of the study.

Blood samples from EC patients were collected between 2004 and 2012. Inclusion criteria for the overall survival (OS) association study were: a diagnosis of EC, availability of complete clinical data, clinical stage ranging from IIB and IVA, survival >3 months after diagnosis, follow-up >24 months for living patients. Differences in patient treatment (neo-adjuvant vs. up-front surgery) was not a discriminant. Patients who died from complication after surgical intervention were excluded. According to these criteria, 180 patients were included in the analysis (Figure 1). Outcome data were collected using clinical or anagraphic records; follow-up was stopped at 108 months; OS was defined as the interval between the date of diagnosis and the date of death from any cause. The 134 patients who had received neoadjuvant treatment, among the 180 patient cohort, were selected for the response association study; all of them completed the therapy cycles and had tumor restaging. Neoadjuvant chemotherapy mainly consisted in treatment with platinum in association with 5-Fluorouracil and concomitant radiation; patients were treated and followed-up exclusively at our Institution. Response criteria referred to RECIST version 1.1. Patients were divided into responder (R: complete and partial response) or non-responder (NR: stable disease and progressive disease). Tumor were staged according to the International Union Against Cancer tumor-node-metastasis (TNM) classification system (7th ed.) and those that were classified with the old edition were re-classified accordingly.

### Genotyping

Genomic DNA was isolated from peripheral blood samples using Flexigene DNA kit (Qiagen Italia, Milano, Italy) following the manufacturer's instructions. DNA concentration and quality were assessed with the NanoDrop 1000 spectrophotometer instrument (Agilent Technologies, Santa Clara, CA, USA). Detection of polymorphism was performed using primers and conditions previously described (25), and applying the Restriction Fragment Length Polymorphism (RFLP)-PCR or the Amplification Refractory Mutation System (ARMS)-PCR method. Positive and negative controls were included in each analysis; re-genotyping of randomly selected samples was 100% concordant.

### Statistical Analysis

Overall survival (OS) curves were estimated using the Kaplan-Meier methods and compared using the log-rank test. Hazard ratios (HRs) and 95% confidence interval were calculated with Cox proportional hazards univariate and multivariate regression models (MedCalc software, v.17.1). Adjustment was made for the following variables: histotype, stage, therapy and age at diagnosis. Association with response to neoadjuvant therapy was calculated by logistic regression, using the SNPStats software (https://www.snpstats.net/start.htm), and adjusted for histotype, stage and age at diagnosis. The receiver operating characteristic (ROC) curves method was used to create and discriminate the predictability of both genetic and clinical models. Comparison of the area under the curve (AUC) values of different ROC curves was performed by the DeLong method (MedCalc software, v.17.1).

Preliminary power estimation to detect association between SNPs and OS was performed using the power of genetic analysis package (26). The minimum detectable effect with odds ratio (OR) was calculated at various allele frequencies of SNP allele under a codominant model (alpha = 0.01; disease prevalence = 1/10,000). With this sample size, we have 80% power to detect an OR of 2.5 if the SNP allele frequency is 40% for single SNP analysis. To account for multiple comparisons (8 tests), the Benjamini and Hochberg false discovery rate (FDR) was used to correct the association with OS, which was our first aim; correction was not applied in the response association tests. *P*-values < 0.05 after correction were considered significant.

## Results

### Patient Cohort for Overall Survival (OS) Association Study

Tumor stage is, together with the response to therapy, one of the more reliable prognostic clinical features; however, a high level of uncertainty and inaccuracy exists for intermediate EC stages. To avoid the confounding effect of clinical stage that could hide the effects of constitutive genetic variants, we excluded from our cohort patients with stage I, IIA and IVB, and restricted the OS association study to 180 patients diagnosed with a clinical stage ranging from IIB to IVA. Table 1 reports the clinico-pathological characteristics of the patients. The median age at diagnosis was 63 years (range 25–86), 147 (82%) were males, adenocarcinoma and squamous carcinoma histotypes were equally represented, stage III was prevalent (73%), 74% received a cisplatin-based neoadjuvant treatment while 26% underwent an up-front surgery. The median survival of the entire cohort was 29.5 months (range 4–108) and the median follow-up for living patients was 66 months (range 28–108). None of the clinical features was statistically associated with the OS.

**Table 1:** Clinico-pathological characteristics of EC patients analyzed for association with overall survival.

**Patients**	**Total**	*P*-value^*^	HR (95%CI)^*^
	**N (%)** **180 (100)**		
**AGE**			
Median (IQR)	63 (56–69)		1
(Range)	(25–86)	0.14	1.01 (0.99–1.03)
**GENDER**			
Male	147 (82)		1
Female	33 (18)	0.39	1.25 (0.75–2.10)
**cSTAGE**			
II (B)	40 (22)		1
III (A-B-C)	131 (73)	0.73	1.53 (0.96–2.46)
IV (A)	9 (5)	0.30	1.55 (0.66–3.61)
**HISTOTYPE**			
EADC	90 (50)		1
ESCC	90 (50)	0.31	1.22 (0.84–1.77)
**TREATMENT**			
Neoadjuvant + surgery	134 (74)		1
Surgery	46 (26)	0.88	0.97 (0.64–1.48)
**OS (MONTH)**			
Median (IQR)	29.5 (14–62)	-	-
(Range)	(4–108)		

* *Cox proportional hazards methods*.

### Genotype Frequencies and Association With OS

All patients were successfully genotyped for rs11615 (Asn118Asn) and rs3212986 (C8092) in the *ERCC1*, and rs13181 (Lys751Gln) and rs1799793 (Asp312Asn) in the *ERCC2/XPD*. Genotype distribution respected the Hardy-Weinberg equilibrium (HWE) for rs11615, rs13181, and rs3212986 with *p*-values ranging from 0.76 to 0.12. On the contrary, rs1799793 in the *ERCC2/XPD* exhibited a *p* = 0.04, suggesting a possible role in EC onset for this genetic variant.

**Figure 2:**
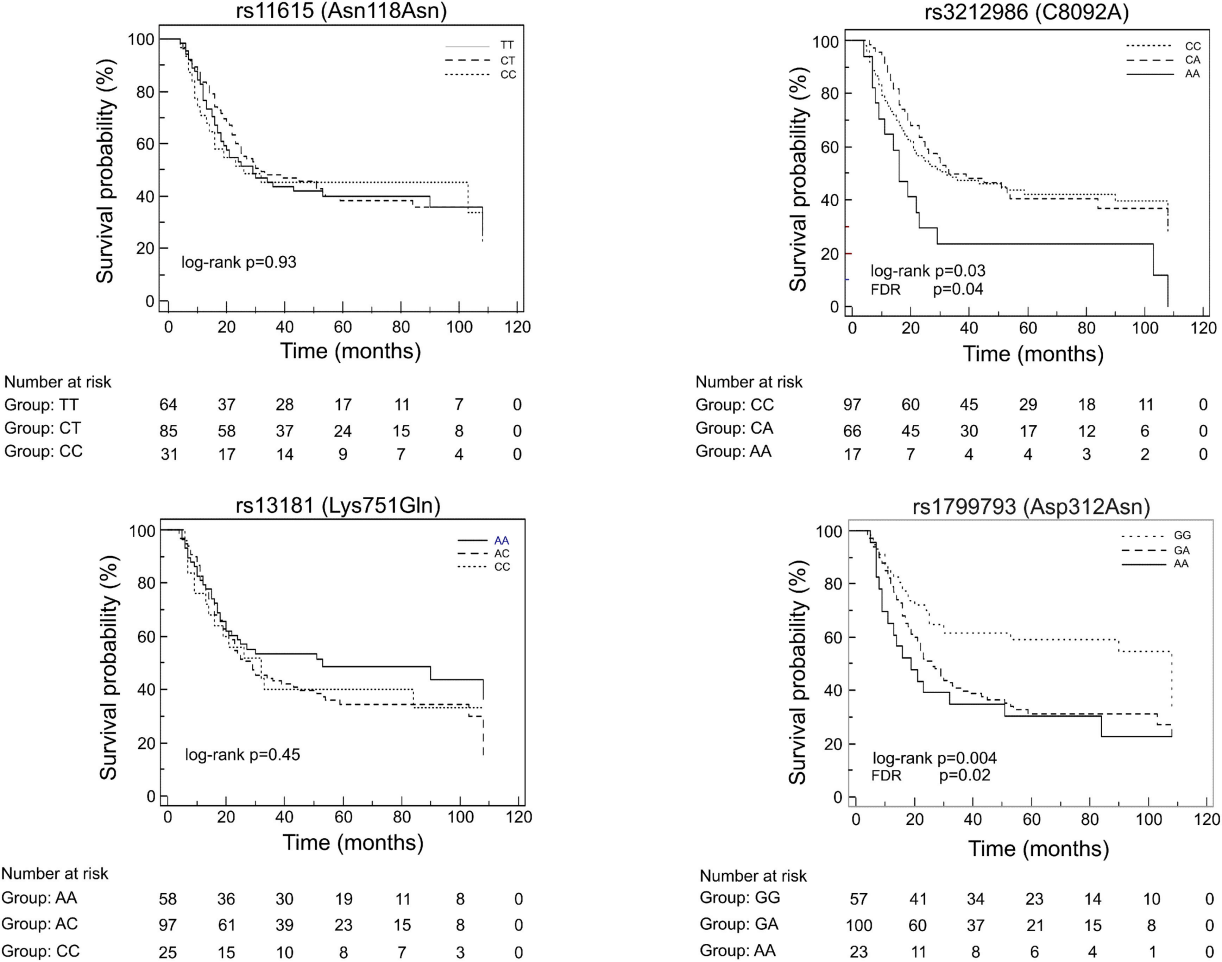
Kaplan-Meier plots of the association between the *ERCC1* rs11615 and rs3212986, and the *ERCC2/XPD* rs13181 and 1799793 and OS.

At first, association between OS and the variants was investigated using the Kaplan-Meier method under codominant genetic model (general model). As reported in Figure 2, among the analyzed variables, only the *ERCC1* rs3212986 and the *ERCC2/XPD* rs1799793 resulted associated with OS (log-rank: *p* = 0.03 and *p* = 0.004, respectively). The median survival of patients carrying the A allele of *ERCC1* rs3212986 in homozygosity was 16 months vs. > 32 months of patients with CC or CA genotypes. Concerning *ERCC2/XPD* rs1799793, the GA and AA genotypes exhibited a median survival of 26 and 19 months, respectively, vs. 47 months of the patients carrying the GG genotype. Comparable results were obtained when survival functions of the *ERCC1* rs3212986 and the *ERCC2/XPD* rs1799793 were analyzed with the univariate Cox proportional method using the codominant model and, based on the Kaplan-Meier plots, the recessive (rs3212986) or the dominant (rs1799793) genetic model (Table 2). Both genetic variants remain statistically associated with survival even after the Benjamini and Hochberg multiple testing correction (Table 2). After adjustment for clinical variables, such as age at diagnosis, histotype, clinical stage, and therapeutic strategy, the association of the *ERCC1* rs3212986 and the *ERCC2/XPD* rs1799793 with OS remained statistically significant (Table 2).

**Table 2:** Univariate and multivariate Cox proportional methods.

			**Univariate**		**FDR**	Multivariate^*^	
**Gene and rsID**	**Genotype**	***N* (%)**	***P*-value**	**HR (95% CI)**	***P*-value**	***P*-value**	**HR (95% CI)**
*ERCC1* (C8092A)	CC	97 (54)		1			1
rs3212986	CA	66 (37)	0.70	0.92 (0.62–1.38)	>1	0.56	0.88 (0.59–1.33)
	AA	17 (9)	0.02	1.93 (1.09–3.40)	0.03	0.06	1.75 (0.98–3.11)
	CC+CA	163 (91)		1			1
	AA	17 (9)	0.01	1.41 (1.08–1.85)	0.02	0.02	1.80 (1.06–3.18)
*ERCC2/XPD* (Asp312Asn)	GG	57 (32)		1			1
rs1799793	GA	100 (56)	0.008	1.8 (1.16–2.84)	0.02	0.02	1.74 (1.11–2.72)
	AA	23 (12)	0.008	2.3 (1.24–4.16)	0.03	0.01	2.19 (1.19–4.03)
	GG	57 (32)		1			1
	GA+AA	123 (68)	0.004	1.89 (1.22–2.92)	0.04	0.007	1.81 (1.17–2.80)

* *Corrected for histological type, clinical stage, treatment, and age at diagnosis*.

### Survival Predictive Model

**Figure 3:**
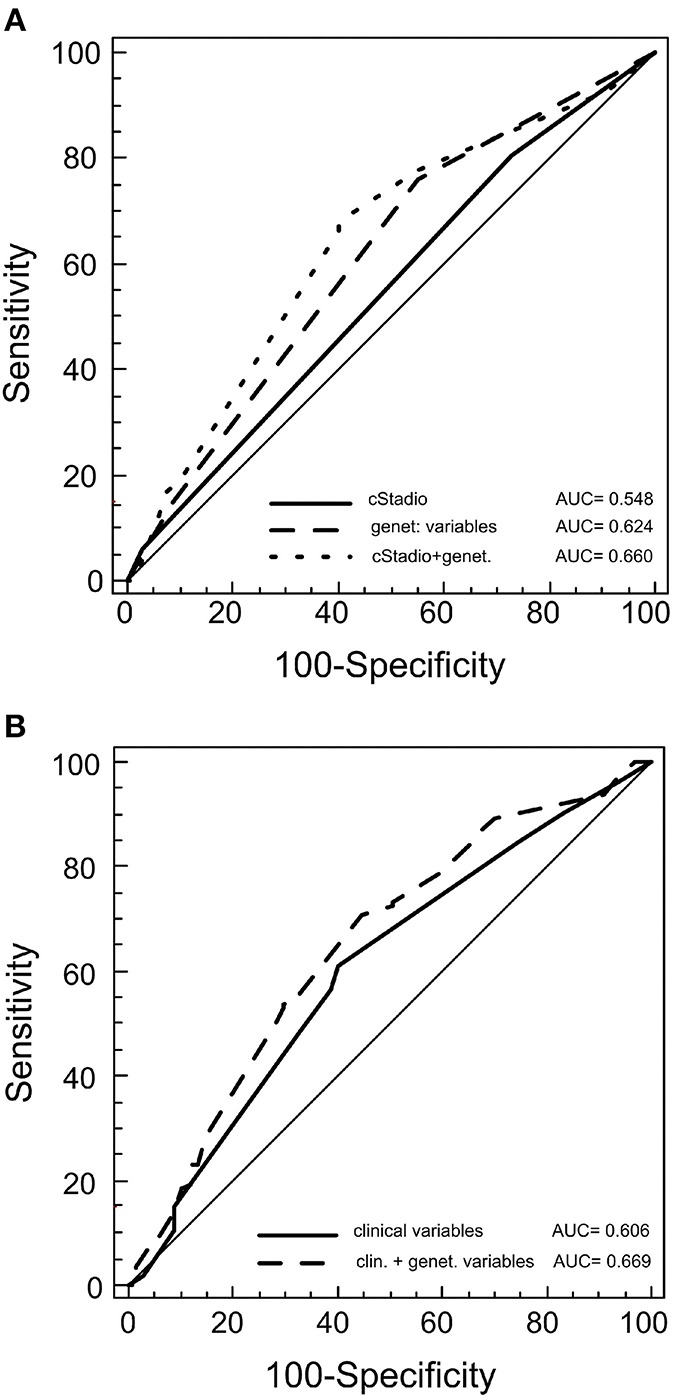
Receiver operating characteristic (ROC) for predicting patient outcome. The plots represent the curves and the area under the curve (AUC) values of the predictive model of germline variants with clinical stage **(A)** or with germline variants with other clinical features (histotype, therapy and stage) **(B)**.

We constructed survival prediction models to explore whether the survival-associated rs3212986 and rs1799793 could increase the predictability of the clinical features. We found that the genetic variants together had a better predictability (area under the curve (AUC) = 0.624) of the clinical stage alone (AUC = 0.548), and of the clinical characteristics (stage, histotype and therapy) together (AUC = 0.606) (Figures 3A,B). When the genetic variables were added to the clinical models, the predictability had a slight increase. This improvement was significant for the clinical stage model: AUC = 0.660, *p* = 0.004 (Figure 3A), but not for the stage, histotype and therapy model: AUC = 0.669, *p* = 0.09 (Figure 3B).

### Patient Cohort for Response Association Study

The second objective of the study was to see whether the same NER variants were associated with response to neoadjuvant therapy. Thus, 134 patients who had had a cisplatin-based neoadjuvant treatment in the total EC cohort were analyzed using the SNPStats software (Figure 1). The clinical and pathological features of this subgroup are reported in Table 3. Median age at diagnosis was 61 years (range 25–80), 81% of patients were males, and the two histotypes were equally represented. All patients received cisplatin-based neoadjuvant therapy and 83% had concomitant radiotherapy. Fifty seven percent of patients were classified as responder (complete and partial response), while 43% were classified as non-responder (patients with stable disease or in progression) following the RECIST 1.1 criteria. No association was observed between response and the other clinical features (Table 3).

**Table 3:** Clinical characteristics of EC patients analyzed for the association with neoadjuvant treatment.

**Patients**	**Total**	*P*-value^*^	OR (95%IC)^*^
	**N (%)** **134 (100)**		
**AGE**			
Median (IQR)	61(55-68)		1
(Range)	(25-80)	0.3	0.99 (0.95–1.02)
**GENDER**			
Male	108 (81)		1
Female	26 (19)	0.5	0.72 (0.29–1.78)
**cSTAGE**			
II (B)	20 (15)		1
III (A-B-C)	105 (78)	0.67	1.23 (0.46–3.29)
IV (A)	9 (7)	0.48	1.80 (0.35–9.39)
**HISTOTYPE**			
EADC	63 (47)		1
ESCC	71 (53)	0.4	1.32 (0.65–2.65)
**NEOADJUVANT**			
CT-RT	111 (83)	0.19	N.A.
CT	23 (17)		
**RESPONSE**			
R	76 (57)	–	–
NR	58 (43)		

* *Logistic Regression*.

### Genotype Frequencies and Association With Response

**Figure 4:**
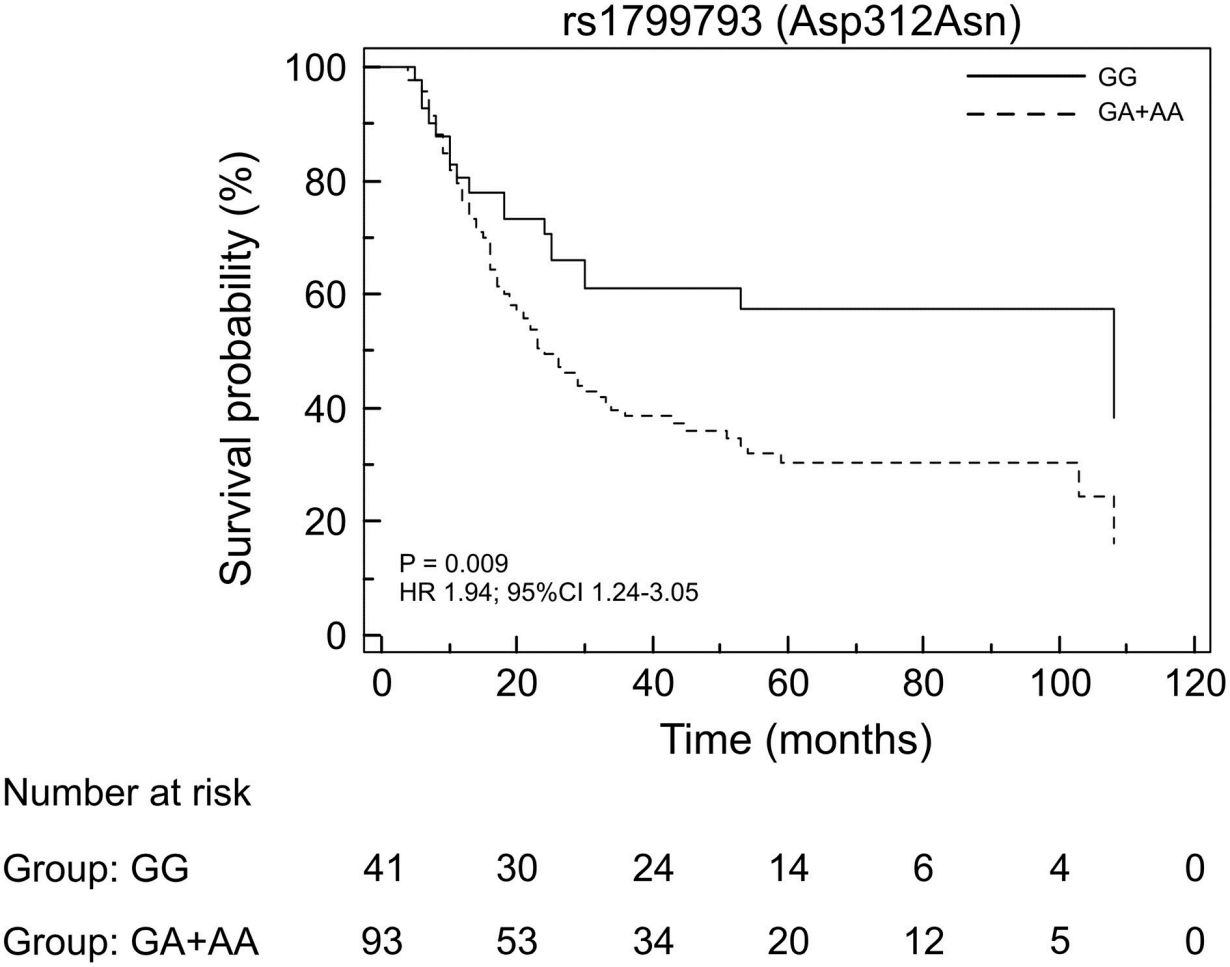
Kaplan-Meier plots of the association of the *ERCC2/XPD* rs1799793 with OS in the EC patients that underwent to neoadjuvant treatment.

By analyzing the 134 EC patients treated with neoadjuvant cisplatin-based chemotherapy, no association with response was found for the *ERCC2/XPD* rs13181 and the *ERCC2/XPD* rs1799793. On the contrary, the minor A allele of *ERCC1* rs3212986 both in homozygosity and heterozygosity exhibited a weak association (*p* = 0.02) as well as the CC genotype of the *ERCC1* rs11615 (*p* = 0.04) (Table 4). Interestingly, the *ERCC2/XPD* rs1799793 though not associated with response (see Table 4), was still associated with OS (*p* = 0.009, under the dominant genetic model) (Figure 4).

**Table 4:** Association analysis of NER pathway genes variants and response to neoadjuvant therapy.

**Gene and rsID**	**Genotype**	**Total 134**	**76 R**	**58 NR**	*p*-value^*^	OR (95%CI)^*^	*p*-value^**^	OR (95%CI)^**^
		***N* (%)**	***N* (%)**	***N* (%)**				
*ERCC1*	TT	44 (33)	27 (35)	17 (29)		1		1
(Asn118Asn)	CT	66 (49)	40 (53)	26 (45)	0.11	1.03 (0.47–2.26)	0.14	1.03 (0.47–2.27)
rs11615	CC	24 (18)	9 (12)	15 (26)		2.65 (0.95–7.38)		2.59 (0.90–7.28)
	TT	44 (33)	27 (35)	17 (29)		1		1
	CT+CC	90 (67)	49 (65)	41 (71)	0.45	1.33 (0.64–2.77)	0.48	1.31 (0.62–2.76)
	TT+CT	110 (82)	67 (88)	43 (74)		**1**		**1**
	CC	24 (18)	9 (12)	15 (26)	**0.04**	**2.60 (1.04–6.46)**	**0.04**	**2.52(1.00–6.36)**
*ERCC1*	CC	69 (51)	46 (61)	23 (40)		1		1
(C8092A)	CA	51 (38)	26 (34)	25 (43)	**0.02**	1.92 (0.91–4.04)	**0.02**	1.80 (0.85–3.83)
rs3212986	AA	14 (11)	4 (5)	10 (17)		**5.00 (1.41–17.68)**		**5.16 (1.42–18.74)**
	CC	69 (51)	46 (61)	23 (40)	**0.02**	**1**	**0.02**	**1**
	CA+AA	65 (49)	30 (39)	35 (60)		**2.33 (1.16–4.69)**		**2.23 (1.10–4.52)**
	CC+CA	120 (89)	72 (95)	48 (83)		**1**		**1**
	AA	14 (11)	4 (5)	10 (17)	**0.02**	**3.75 (1.11–12.65)**	**0.02**	**4.00 (1.15–13.90)**
*ERCC2/XPD*	AA	41 (31)	24 (32)	17 (29)		1		1
(Lys751Gln)	AC	77 (57)	44 (58)	33 (57)	0.84	1.06 (0.49–2.28)	0.84	1.07 (0.49–2.34)
rs13181	CC	16 (12)	8 (11)	8 (14)		1.41 (0.44–4.51)		1.42 (0.44–4.59)
	AA	41 (31)	24 (32)	17 (29)		1		1
	AC+CC	93 (69)	52 (68)	41 (71)	0.78	1.11 (0.53–2.34)	0.76	1.13 (0.53–2.40)
	AA+AC	118 (88)	68 (89)	50 (86)		1		1
	CC	16 (12)	8 (11)	8 (14)	0.56	1.36 (0.48–3.87)	0.57	1.36 (0.47–3.90)
*ERCC2/XPD*	GG	41 (31)	28 (37)	13 (23)		1		1
(Asp312Asn)	GA	77 (57)	42 (55)	35 (60)	0.09	1.79 (0.81–3.89)	0.09	1.82 (0.81–4.10)
rs1799793	AA	16 (12)	6 (8)	10 (17)		3.59 (1.07–12.0)		3.65 (1.07–12.43)
	GG	41 (31)	28 (37)	13 (23)		1		1
	GA+AA	93 (69)	48 (63)	45 (77)	0.07	2.02 (0.93–4.38)	0.07	2.05 (0.93–4.51)
	GG+GA	118 (88)	70 (92)	48 (83)		1		1
	AA	16 (12)	6 (8)	10 (17)	0.1	2.43 (0.83–7.13)	0.10	2.43 (0.82–7.23)

* *Logistic regression*.

** *Adjuasted for hystotype, stage and age at diagnosis*.

*statistically significant values are reported in bold*.

## Discussion

Although tumor stage and resection status are the main prognostic factors in EC, other reliable biomarkers that provide a better prediction of outcome, especially for the advanced stages, are still needed. Germline variants in DNA repair pathway genes have been extensively studied to check their influence in tumor onset, response to chemotherapy and OS. However, inconsistent results were obtained, with some studies highlighting the relevance of some genetic variants and others emphasizing opposite results (20, 21, 24, 27).

In this study, we investigated the association between four germline variants in DNA repair genes, two in the *ERCC1* (rs11615 and rs3212986), and two in the *ERCC2/XPD* (rs13181 and rs1799793) and the outcome of patients with advanced EC. ERCC1 and ERCC2/XPD are considered two pivotal proteins of the NER pathway. The ERCC1 protein is responsible for the recognition and excision of the damaged DNA while the *ERCC2/XPD* codes for a helicase that, besides being a major player in DNA repair, is also a component of transcription factor II H (TFIIH) complex (18, 28, 29).

We observed that, among the analyzed variants, the *ERCC1* rs3212986 and the *ERCC2/XPD* rs1799793 remained statistically associated with a poor OS after correction for multiple tests. Patients carrying the minor A allele of the rs3212986 in homozygosity had a median survival of 16 months vs. > 32 months in patients homozygote or heterozygote for the major C allele. Concerning the rs1799793, patients carrying the variant A allele both in homozygosity or heterozygosity exhibited a significant shorter survival compared to patients with the GG genotype (median survival 23 months vs. 47 months). Adjustment with clinical features did not change the significance suggesting that they are independent prognostic biomarkers. When, as a second aim, we analyzed the association of the same NER variants with the response to neoadjuvant therapy, only the rs3212986 and rs11615 in *ERCC1* exhibited an association. Interestingly, both *ERCC2/XPD* variants were not associated. This last finding strengthens the hypothesis that *ERCC2/XPD* rs1799793 may only have a prognostic value.

Association of *ERCC1* rs3212986 and *ERCC2/XPD* rs1799793 with survival has been previously investigated in a few EC Caucasian patient cohorts and results are in contrast with those reported in this study (24). In particular, Bradbury and co-workers reported that the presence of the CA and AA genotypes of the rs3212986, and the GA and AA genotypes of the rs1799793 correlated with a better outcome (30). Similarly, in a previous study conducted in a small cohort of Caucasian EADC and ESCC patients, we also found a positive association between OS and the minor allele of *ERCC1* rs3212986 (25). In the same cohort, no association between survival and rs1799793 was found. We believe that the inclusion of a broad range of different tumor stages (from early to metastatic) in the work by Bradbury et al, and the small sample size analyzed in our previous study are the major cause of these discrepancies. Indeed, owing to the relatively low hazard risk of each single germline variant, the samples homogeneity and size are the most critical points in pharmacogenetic studies.

The observed deviation from HWE of the *ERCC2/XPD* rs1799793 in our cohort might reflect its involvement in development of the EC (31, 32). The contribution of rs1799793 to EC onset might also explain its impact in patient survival by promoting tumor progression and aggressiveness, rather than by influencing the response to therapeutic treatment.

So far, the altered DNA repair activity of the ERCC1 and ERCC2/XPD variants has been considered central for its association with tumor risk and cancer patient outcome (17, 19). However, ERCC2/XPD is a helicase also endowed with transcriptional activity, and the codon Asp312Asn is located in the Arch transcriptional domain (that encompasses codons S246-D439) of the protein. Thus, it is conceivable that this germline amino acid substitution might principally affect its transcriptional activity, rather than DNA repair, as reported for mutations located in this domain (33, 34). Furthermore, this possibility might also explain why the rs1799793 influences patient survival but not the efficacy of chemotherapy, which is considered more related to the removal of DNA adducts.

Although our data clearly indicate that the *ERCC1* rs3212986 and the *ERCC2/XPD* rs1799793 affect OS in advanced EC, the study presents some limitations. Besides its retrospective nature, other constraints are the limited number of SNPs analyzed and the candidate gene approach. This can explain the poor clinical impact of the rs3212986 and the rs1799793 (AUC 0.669) as prognostic biomarkers, despite their statistical significant association with OS. Indeed, although there is no doubt about the relevance of constitutive variants on cancer onset and outcome, their limited power is still the greatest obstacle to their clinical use. It has been suggested that the restricted influence of germline SNPs can be overcome by enlarging the pharmacogenetic analyses to additional variants and by calculating the overall risk (i.e., polymorphic risk score) (35–37). Thus, further studies are needed to find other germline variants that, together with the rs3212986 and the rs1799793, could generate a prognostic panel with increased clinical impact. Nevertheless, until the discovery of a more powerful prognostic signature, we believe that both the *ERCC1* rs3212986 and the *ERCC2/XPD* rs1799793 could contribute to the better stratification of patients with advanced resectable EC.

## Ethics Statement

The study had the approval of the Comitato Etico per la Sperimentazione Clinica (CESC) of the Veneto Institute of Oncology IOV-IRCCS (cod. number CE IOV 2012/65), and was carried out according to the Code of Ethics of the World Medical Association (Declaration of Helsinki). All patients gave written informed consent in accordance with the declaration of Helsinki.

## Author Contributions

EB, SM, ER, and DS provided the concept and design of the study. SM, ER, GB, and AR participated to the acquisition of data, analysis, and interpretation. EB, SM, and DS analyzed and interpretated the data, drafted the article. AA and AR critically revised the manuscript.

### Conflict of Interest Statement

The authors declare that the research was conducted in the absence of any commercial or financial relationships that could be construed as a potential conflict of interest.
